# Online Health Information Seeking Behaviors of Hispanics in New York City: A Community-Based Cross-Sectional Study

**DOI:** 10.2196/jmir.3499

**Published:** 2014-07-22

**Authors:** Young Ji Lee, Bernadette Boden-Albala, Elaine Larson, Adam Wilcox, Suzanne Bakken

**Affiliations:** ^1^Division of Health and Biomedical InformaticsDepartment of Preventive MedicineNorthwestern UniversityChicago, ILUnited States; ^2^Global Institute of Public HealthNew York UniversityNew York, NYUnited States; ^3^School of NursingColumbia UniversityNew York, NYUnited States; ^4^Department of EpidemiologyMailman School of Public HealthColumbia UniversityNew York, NYUnited States; ^5^Intermountain HealthcareMurray, UTUnited States; ^6^Department of Biomedical InformaticsColumbia UniversityNew York, NYUnited States

**Keywords:** Internet, information seeking behavior, health behavior, consumer health information, hispanic Americans

## Abstract

**Background:**

The emergence of the Internet has increased access to health information and can facilitate active individual engagement in health care decision making. Hispanics are the fastest-growing minority group in the United States and are also the most underserved in terms of access to online health information. A growing body of literature has examined correlates of online health information seeking behaviors (HISBs), but few studies have included Hispanics.

**Objective:**

The specific aim of this descriptive, correlational study was to examine factors associated with HISBs of Hispanics.

**Methods:**

The study sample (N=4070) was recruited from five postal zip codes in northern Manhattan for the Washington Heights Inwood Informatics Infrastructure for Comparative Effectiveness Research project. Survey data were collected via interview by bilingual community health workers in a community center, households, and other community settings. Data were analyzed using bivariate analyses and logistic regression.

**Results:**

Among individual respondents, online HISBs were significantly associated with higher education (OR 3.03, 95% CI 2.15-4.29, *P*<.001), worse health status (OR 0.42, 95% CI 0.31-0.57, *P*<.001), and having no hypertension (OR 0.60, 95% CI 0.43-0.84, *P*=.003). Online HISBs of other household members were significantly associated with respondent factors: female gender (OR 1.60, 95% CI 1.22-2.10, *P*=.001), being younger (OR 0.75, 95% CI 0.62-0.90, *P*=.002), being married (OR 1.36, 95% CI 1.09-1.71, *P*=.007), having higher education (OR 1.80, 95% CI 1.404-2.316, *P*<.001), being in worse health (OR 0.59, 95% CI 0.46-0.77, *P*<.001), and having serious health problems increased the odds of their household members’ online HISBs (OR 1.83, 95% CI 1.29-2.60, *P*=.001).

**Conclusions:**

This large-scale community survey identified factors associated with online HISBs among Hispanics that merit closer examination. To enhance online HISBs among Hispanics, health care providers and policy makers need to understand the cultural context of the Hispanic population. Results of this study can provide a foundation for the development of informatics-based interventions to improve the health of Hispanics in the United States.

##  Introduction

Since the Internet has become a promising source of health information for the general public and a target of health information seeking behaviors (HISBs), the use of the Internet for health purposes is an important topic [1-6]. Those living in metropolitan areas with populations larger than 250,000 have been more likely to access the Internet than residents of rural communities [7-9]. Moreover, individuals residing in urban areas are more likely to use the Internet to seek health-related information [3,9]. Although use of the Internet differs by geographic location, social and economic disparities such as geographic distribution of race and ethnicity groups likely account for those differences [9,10].

Hispanics are the most underserved population in the US in terms of access to online health information because of limited opportunities for education and employment and an increased prevalence of poverty [10-13]. According to the US Census Bureau, 16% of the US population (50.5 million) identified themselves as Hispanic or Latino in 2010 [14]. Of Hispanic Internet users, 66% have used the Internet for searching health information. However, non-Hispanic whites and African Americans are more likely to seek health information through the Internet: 73% and 69% respectively [15].

Reducing the inequality of access to health information is linked to reducing and preventing an unequal burden of disease. In 2010, the Department of Health and Human Services launched “Healthy People 2020”, which included an objective “to increase the proportion of online health information seekers who report that they can easily access health information” [16]. To meet this objective, the characteristics of individuals who search for health information via the Internet needed to be identified [17,18]. A growing body of literature has examined correlates of online HISBs; however, to our knowledge, there are few such studies about the Hispanic community.

As a part of a larger study, the Washington Heights Inwood Informatics Infrastructure for Comparative Effectiveness Research (WICER) project, our study aimed to examine factors associated with online HISBs among Hispanics.

##  Methods

### Theoretical Framework

Bodie and Dutta’s Integrative Model of eHealth Use [19] informed the development of the research question and selection of study variables for our study: What demographic, situational, and literacy factors (health and computer literacy) are associated with online HISBs among Hispanic survey respondents and other members of the same household?

According to the model, variables such as demographics, situational, personal, and cultural factors affect the use of the Internet for obtaining health information [19]. Differences in these variables may contribute to health disparities and a digital divide between people who have and people who do not have access to Internet technology [19]. This model suggests that disparities in social structures such as socioeconomic factors lead to individual-level differences in motivation and online health information seeking ability. The difference in online HISBs causes disparities in lifestyle that are related to health outcomes and continue to contribute to health care disparities [19].

### Setting and Sample

The study setting included five zip codes (10031, 10032, 10033, 10034, and 10040) that represent the Washington Heights Inwood community of Northern Manhattan. These communities have been designated as medically underserved areas by the Centers for Medicare and Medicaid Services since they meet the relevant criteria regarding the level of poverty, the proportion of elderly, the incidence of infant mortality, and the ratio of primary care providers to population [20,21]. Currently, 71% of Washington Heights and Inwood area residents are Hispanic [22]. The sample comprised 4070 residents, who completed the WICER household survey between March 2011 and November 2012. Residents who were 18 years or older, English or Spanish speaking, and Hispanic were eligible for inclusion in the study.

### Recruitment

After approval by the Columbia University Medical Center Institutional Review Board, recruitment of eligible participants was initiated using multiple methods. Data were collected in households, businesses, or at a designated community space, the Columbia-Community Partnership for Health (CCPH). For the CCPH sample, we recruited a convenience sample of individuals who came to the Center for blood pressure checks or because they were referred by friends. Most of the participants were recruited by a snowball sampling method using respondents’ social networks. At the end of the survey interview, interviewers asked participants if they were willing to refer members of their social network for study participation.

###  Survey Procedures

All interviewers were bilingual and familiar with the Washington Heights and Inwood community. They completed relevant human subjects research training and didactic and field training with the interview guide. Their work was monitored on a daily basis by the survey coordinator who reviewed all interview data. The survey coordinator also conducted regular spot checks of the data collection process in the field. Interviewees were re-trained on an as-needed basis. Before conducting interviews, the trained interviewer obtained informed consent from the participant in their language of choice (English or Spanish). All survey items were self-reported. The interview process took approximately 45 minutes to one hour, and participants received US $25 compensation for their time.

### Data Management

All survey data were entered into Lime Survey, a Web-based data management tool, on a secure server. The baseline survey from unique respondents was used in the analyses. Data were cleaned, and subjects with missing or invalid values for the study variables were removed from the analysis. Data were extracted from Lime Survey into SPSS v. 20.0 for analysis.

### Study Variables

Based upon the theoretical framework for the study, the correlates of interest in this study were demographic information, situational factors, health literacy, and computer literacy (Table 1). The two dependent variables in the study were online HISBs of respondents and online HISBs of their household members.

**Table 1:** Conceptualization and measurement of study variables.

Concept	Variable	Definition	Data type	Instrument
Demographic information	Age, gender, employment, marital status, educational level, insurance		Categorical, Continuous	
Situational factors	Hypertension, health problems, general health status	The specific health situations faced by a patient and their subsequent consumer health information needs	Categorical	Blood pressure question [23], Chronic Burden Scale [24], SF-8 health survey [25]
Health literacy	Health literacy	The degree to which individuals have the capacity to obtain, process, and understand basic health information and services needed to make appropriate health decisions	Continuous	Newest Vital Sign English or Spanish [26]
Computer literacy	Experiences of social networking	Computer skills and ability to use technology to improve learning, productivity, and performance	Categorical	Use of social networking sites
Online health information seeking behaviors	Online support group or communication with clinicians	The interaction of an individual with or through an electronic device or communication technology to access or transmit health information or to receive guidance and support on a health-related issue	Categorical	Health Information National Trends Survey (HINTS) [27]

### Instruments

#### Demographic and Situational Factors

As summarized in Table 1, demographic and situational data regarding the household respondent and each household member were obtained during the interview. Hypertension was measured by the question, “Have you ever been told by a doctor, nurse, or other health professional that you had hypertension also called high blood pressure or pressure?” [23]. Serious health problems were measured by a question, “Have you experienced any serious personal health problems that have lasted for at least 6 months?” from the Chronic Burden Scale [24]. Self-reported general health status was recorded in five categories: excellent, very good, good, fair, and poor. General health status was measured on a 5-point Likert scale (1=excellent and 5=poor) from the Short Form-8 Health Survey (SF-8) [25].

#### Health Literacy

The Newest Vital Sign in either English or Spanish (NVS) was used to assess health literacy in the study population. The NVS includes 6 questions to test reading, interpretation, and numeracy skills based on a nutritional label from an ice cream container [26]. This general measure was selected, rather than a more specific measure of eHealth literacy, because of its wide use and WICER’s overall goal of understanding the health of the community and social determinants influencing health.

#### Computer Literacy

The US Department of Education defines computer literacy as “computer skills and ability to use technology to improve learning, productivity, and performance” [28]. However, the definition of computer literacy changes with the technology evolution [29], and several recent studies have demonstrated the positive relationship between use of social networking sites and computer literacy [30,31].

Based on this evidence and the fact that computer literacy was not directly measured in the WICER study, use of social network sites was used as a proxy measure for computer literacy. It was measured by the question, “Do you belong to any social networking sites like Facebook, MySpace, or Twitter?

#### Online Health Information Seeking Behaviors

Robinson et al (1998) defined interactive health communication as “the interaction of an individual—consumer, patient, caregiver or professional—with or through an electronic device or communication technology to access or transmit health information or to receive guidance and support on a health-related issue” [32]. Based on the definition, this study considered participation in an online support group, email communication with physicians, and using the Internet to look up health or medical information as online HISBs in this study.

Thus, to measure online HISBs, four questions from the Health Information National Trends Survey (HINTS) were used [27]. Respondents’ HISBs were measured using three questions: “In the past 12 months, (1) Have you participated in an online support group for people with similar health or medical issues? (2) Have you used email or the Internet to communicate with a doctor or doctor’s office? (3) Have you used the Internet to look up health or medical information?” The HISBs of respondents’ household members were measured using a single question: (4) “Does anyone in your household use the Internet to look up health or medical information?” An affirmative response to any of the first three questions was coded as “yes” on online HISBs. An affirmative response to the fourth question was coded as “yes” on household HISBs.

### Statistical Analysis

Respondents’ online HISBs and those of other household members were analyzed separately. Education was coded as < or ≥ high school, insurance as yes/no, and birthplace as United States or elsewhere. Health literacy scores from NVS were used as a continuous variable [33]. For the NVS, “refused” or “don’t know” and missing values were treated as wrong answers and received 0 points.

Initially, univariate analyses were used to examine the frequency and distribution of study variables, calculating mean and standard deviation, range, frequency, and percentage as appropriate. Correlates of respondents’ HISBs and those of respondents’ household members were analyzed in separate models. Bivariate analyses, including chi-square tests and *t* tests, were used to examine differences between those reporting HISBs and those who did not (separately for respondents and for their household members) in terms of demographic, situational, and literacy (health, computer) variables.

Binary logistic regression analyses were conducted to examine factors associated with online HISBs of respondent and online HISBs of household members. Variables that were significant in the chi-square or *t* test were selected for regression models and entered hierarchically with demographic and situational factors entered first followed by literacy factors. Level of significance for testing of each model was set to an alpha of .05.

##  Results

### Characteristics of Respondents

#### Demographic Factors

The average age of respondents was 51.2 years old (SD 16.8, range 18-104); 71.33% (2903/4070) were women and 88.80% (3614/4070) were foreign born (Table 2). More than half of the respondents were unemployed (65.18%, 2653/4070), not married (63.10%, 2568/4070), and had a less than high school education (51.74%, 2106/4070). A majority of participants (77.00%, 3134/4070) were Medicare or Medicaid beneficiaries, 15.33% (624/4070) had private or other insurance, and 8.79% (358/4070) were uninsured.

**Table 2:** Descriptive characteristics of Hispanic participants (N=4070).

Variables			Respondents, n (%)
**Demographic factors**			
	**Gender**		
		Men	1133 (27.84)
		Women	2903 (71.33)
	**Employment status**		
		Employed	1411 (34.67)
		Unemployed	2653 (65.18)
	**Marital status**		
		Married/living as	1457 (35.79)
		Otherwise	2568 (63.09)
	**Education**		
		<High school graduate	2106 (51.74)
		≥High school graduate	1906 (46.83)
	**Birthplace**		
		Born in the United States	445 (10.93)
		Born in other countries	3614 (88.79)
	**Insurance**		
		Medicare/Medicaid	3134 (77.00)
		Others (veterans, private, etc)	624 (15.33)
		None	358 (8.79)
**Situational factors**			
	**General health status**		
		<Good	921 (22.63)
		≥Good	3055 (75.06)
	**Hypertension**		
		Yes	1608 (39.50)
		No	2426 (59.60)
	**Serious personal health problems**		
		Yes	342 (8.40)
		No	3696 (90.81)
**Literacy factors**			
	**Social networking sites**		
		Yes	867 (21.30)
		No	2815 (69.16)
**Online health information seeking behaviors**			
	Respondent		317 (7.89)
	Household member		466 (11.45)
**Demographic factors**			
	Age, mean (SD)		51.2 (16.81)
**Literacy factors**			
	Health literacy, mean (SD)		2.1 (1.96)

#### Situational Factors

Most respondents reported their general health status as at least “good” (75.06%, 3055/4070) and without serious health problems (90.81%, 3696/4070). A large proportion of respondents (39.51%, 1608/4070) answered that they had been diagnosed with hypertension by a clinician.

#### Literacy Factors

The mean NVS score was 2.2 (SD 1.96), indicating the possibility of marginal or inadequate literacy. For computer literacy, only 21.30% of respondents (867/4070) answered that they had used social networking sites.

#### Online Health Information Seeking Behaviors

Only 7.79% of respondents (317/4070) reported at least one of three HISBs (ie, online support group, email communication with physician, used the Internet to search for health-related information); 11.45% (466/4070) reported that a household member had used the Internet to search for health-related information.

### Factors Associated With Online Health Information Seeking Behaviors

#### Primary Respondent

Several demographic and situational factors were independently associated with respondent online HISBs, including having higher education (OR 3.03, 95% CI 2.15-4.29, *P*<.001), being in worse health status (OR 0.42, 95% CI 0.31-0.57, *P*<.001), and having no hypertension (OR 0.60, 95% CI 0.43-0.84, *P*=.003). Social networking site users were more than three times more likely than non-users to seek health information online (OR 3.78, 95% CI 2.78- 5.13, *P*<.001) (Table 3). Model fit was poor (Hosmer and Lemeshow χ^2^_8_=17.78, *P*=.02).

**Table 3:** Binary logistic regression: correlates of respondents’ online HISBs (N=4070).

Respondent factors		Model OR (95% CI)	*P* value
**Demographic and situational factors**			
	Gender	1.33 (0.96-1.83)	.08
	Age	1.14 (0.92-1.41)	.25
	General health status	0.42 (0.31-0.57)^a^	<.001
	Hypertension	0.60 (0.43-0.84)^b^	.003
	Insurance	0.81 (0.54-1.22)	.31
	Employment status	1.17 (0.88-1.54)	.29
	Education level	3.03 (2.15-4.29)^a^	<.001
	Nativity	1.25 (0.88-1.77)	.22
**Literacy factors**			
	Health literacy level (NVS)	0.99 (0.93-1.07)	.87
	Use of SNS	3.78 (2.78-5.13)^a^	<.001
Hosmer and Lemeshow χ^2^_8_		17.78^c^	.02

^a^ *P*<.001.

^b^ *P*<.01.

^c^ *P*<.05.

#### Other Household Members

Respondents’ use of social networking was a significant factor (OR 2.24, 95% CI 1.74-2.89, *P*<.001), controlling for other factors, in predicting household members HISBs. Additionally, several respondents’ characteristics were associated with their household members online HISBs: female (OR 1.60, 95% CI 1.22-2.10, *P*=.001), younger (OR 0.75, 95% CI 0.62-0.90, *P*=.002), married (OR 1.36, 95% CI 1.09-1.71, *P*=.007), higher education (OR 1.80, 95% CI 1.404-2.316, *P*<.001), being in worse health status (OR 0.59, 95% CI 0.46-0.77, *P*<.001), and having serious health problem (OR 1.83, 95% CI 1.29-2.60, *P*=.001) (Table 4). The model for household members’ online HISBs demonstrated good fit (Hosmer and Lemeshow χ^2^_8_=6.31, *P*=.66).

**Table 4:** Binary logistic regression: correlates of household members’ online HISBs (N=4070).

Respondent factors		Model OR (95% CI)	*P* value
**Demographic and situational factors**			
	Gender	1.60 (1.22-2.10)^a^	.001
	Age	0.75 (0.62-0.90)^a^	.002
	General health status	0.59 (0.46-0.77)^b^	<.001
	Hypertension	0.93 (0.72-1.21)	.60
	Marital status	1.36 (1.09-1.71)^a^	.007
	Employment status	1.18 (0.94-1.49)	.16
	Education level	1.80 (1.40-2.32)^b^	<.001
	Serious health problem	1.83 (1.29-2.60)^a^	.001
**Literacy factors**			
	Health literacy level (NVS)	0.97 (0.91-1.03)	.26
	Use of SNS	2.24 (1.74-2.90)^b^	<.001
Hosmer and Lemeshow χ^2^_8_		5.85	.66

^a^ *P*<.01.

^b^ *P*<.001.

## Discussion

### Principal Results

#### Summary

Most Hispanics have sought health information from family and friends or community groups in the past year [34]. Recently, there is increased reliance on the Internet. The National Health Interview Survey conducted by National Center for Health Statistics showed that 28.8% of Hispanics aged 18-64 used the Internet to find health-related information [35]. However, only 7.79% of our survey respondents reported online HISBs, which is significantly lower than the national data [35]. There are several potential reasons for this difference. First, Washington Height and Inwood are designated as medically underserved areas. Another study conducted in a medically underserved area showed that only 21% of respondents accessed to the Internet for health information [21]. This suggests that there may be a significant disparity in online HISBs between underserved populations and the general population [21]. Second, there may also be inequalities in access to online health information within Hispanics due to the heterogeneity of that population [11,26,36,37], which may explain differences between our findings and the national data.

This study provides new data regarding correlates of the use of the Internet by Hispanics for seeking health information. Findings are summarized and then discussed according to the concepts from the Integrative Model of eHealth Use [19].

Higher education level, being in worse (poor/fair) health status, having normal blood pressure (ie, no hypertension), and being computer literate were positively associated with online HISBs. However, the final model of respondents’ online HISBs demonstrated poor fit. Given the relatively large sample size of this study (N=4070), small differences across the sample can influence model fit [38]. Thus, this is one potential reason for the poor model fit. Other potential reasons for the poor model fit are missing variables that are associated with online HISBs and operationalization of the study measures.

Furthermore, respondents’ demographic, situational, and computer literacy factors were associated with increased odds of their household members’ online HISBs. Household members were more likely to seek health-related information through the Internet when the respondents were female, younger, married, highly educated, computer literate, in worse health status, and had serious health problems. In contrast to the respondent model, this model demonstrated good fit with the data.

#### Demographic Characteristics

Studies of the general population in the United States have shown that being female [4,39-43], being younger [4,7,40,43-47], and having more education are positively associated with online HISBs [3,4,7,18,39-41,44-49]. Studies on Hispanics have shown similar results [11,50]. Our findings are consistent with previous studies that showed that better-educated respondents were more likely to access health information through the Internet. In particular, Miller et al found that Hispanics had the strongest relationship between education and online HISBs among ethnic and racial groups [51].

However, some of the findings were inconsistent with those reported in the literature. Age did not influence respondents’ online HISBs in our study, whereas earlier studies showed that younger age is positively related to online HISBs [4,7,40,43-47,50]. This may be because the great majority of the respondents were over 40 years old. Among the survey respondents, 20.74 % were over 65 years old (844/4070), and 51.33% of respondents were between 40 and 64 years old (2089/4070). Recently, the older adults who use the Internet as a source of health information have been increasing. About 69% of the population over age 65 report online HISBs [52,53]. The number will continue to increase, since the majority of online health information seekers are adults between 40 and 59 years old [53,54]. In addition, the US census showed that among Internet users, older people are more engaged in online HISBs than younger people [55].

In contrast to our findings, previous studies showed that females were more likely to search health information through the Internet [4,11,39-43]. One study found that men and people without children were more likely to seek health information for themselves rather than others [56]. The relatively small proportion of males (27.84%, 1133/4070) coupled with the low online HISB may have influenced the ability to detect gender differences even in our large sample. However, our findings suggest that respondents’ gender and marital status are associated with household members’ online HISBs. Women play a key role in managing the health of the Hispanic family [11]; for example, household members may seek health information at a female family member’s request. The relationship between respondents’ marital status and their household members’ online HISBs is consistent with Sadasivam’s finding that being married was positively associated with surrogate-seekers’ online HISBs [57]. In that study, a surrogate seeker was defined as a person who looked for health information for family members or friends [57].

Respondents’ higher education level was positively associated with household members’ online HISBs. Recent studies have shown that children of less-educated parents are less likely to seek health information [58]. In our study, about one third of household members who had sought health-related information were sons, daughters, or grandchildren 33.9% (158/466) of the respondent.

#### Situational Factors

Our study showed that individuals with poor health status were more likely to seek health information through the Internet. This is consistent with previous studies that have found that people with poor health status may have stronger needs for information [59,60]. Online health information can meet their higher demand for health information because of easy access. Their needs for health information may lead them to use online health information to manage their health [60].

Respondents without hypertension (ie, normal blood pressure) were more likely to seek health information through the Internet. Previous studies have found that having chronic disease including hypertension was positively associated with online HISBs [48,61]. However, Ayers and Kronenfeld suggested that online HISBs are not merely affected by the presence of a particular chronic illness, but rather by the total number of chronic conditions [62]. Furthermore, several studies have found that individuals who have hypertension seek health information less than those with other chronic diseases [54]. A Pew Internet survey showed that among the online health information seekers with one or more chronic conditions, the percentage of hypertension patients (57%) was less than that of cancer patients (62%) or lung patients (68%) [63]. These findings provide a possible rationale for the association between hypertension status and online HISBs.

For the household members, it is not possible to determine from this study if the household member looked up health information for themselves, the respondents, or another friend or family member. Household members may have sought health information for respondents since respondents suffered from serious health problems and they perceived their health status as poor. Familism, an important Hispanic cultural value with implications for the engagement of family members in the care of a patient, is a possible rationale for this finding [11,36]. Furthermore, most survey respondents were immigrants. Among immigrants, the family plays an important role in HISBs. Instead of consulting with health care providers, they often ask their family members about health information and for advice [64,65].

#### Literacy Factors

An individual who is computer literate is more likely to go to the Internet for finding health information. Several studies have shown that the ability to use a computer is related to online HISBs [66]. In our study, computer literacy as measured by social networking was positively associated with online HISBs. Moreover, respondents’ computer literacy was also positively associated with household members’ online HISBs. This is consistent with a study that found that parents’ computer literacy may affect children’s computer use [67]. Respondents in our study answered that 33.9% of their household members who went to the Internet for health information were their children.

Health literacy was not significantly associated with online HISBs of respondents or their household members, although it has consistently been identified in the literature as a challenge when people use the Internet to search for health information [68-71]. A possible explanation for the lack of significance is the floor effect [33] because most respondents scored low on the NVS.

### Limitations

There are several limitations to this study. Generalizability is a potential limitation of this study due to the non-probability sampling method. Although the study adopted several sampling methods for recruitment, most participants were recruited using non-probability sampling, and the resulting sample is more Hispanic, female, and older than the Washington Heights and Inwood population. Moreover, this study recruited Hispanics who lived in urban areas in New York City; therefore, the findings may not be generalizable to Hispanics living in rural areas or in other cities.

Second, because our study relied on self-reported information, social desirability is a potential concern. Sometimes respondents tend to answer in a way that they think the researcher wants. They tend to over-report for the desired behaviors such as physical activity and under- report undesirable ones such as alcohol consumption [72]. Furthermore, the question regarding household members’ online HISB was answered by respondents on behalf of household members. Therefore, it is possible that the percentage of online HISBs among household members may not be accurate.

Third, this study did not explicitly identify respondents who accessed the Internet from their cell phones. A Pew Hispanic Center reported that Hispanics are more likely than non- Hispanics to access the Internet through mobile devices; 76% of Hispanics access the mobile Internet compared to 60% of non-Hispanics [73]. Therefore, this study may have underestimated online HISBs among the survey respondents. An alternative explanation is that our use of bilingual data collectors resulted in a sample that included individuals not typically included in other studies.

### Implications

Online HISBs can lead patients to make informed health care decisions by increasing their participation in health management. Those decisions may affect the relationship with health care providers [62]. To assist patients, there is a need for a health information infrastructure for shared decision making between patients and the health care system [74]. However, decision-making interventions may not be successfully implemented if discrepancies exist across populations [75]. Our study of online HISBs among Hispanics may suggest what needs to be considered to resolve the discrepancy and to implement an infrastructure in Hispanic community such as the one that we studied.

However, we showed that overall access for online health information was lower than national data. Government agencies and policy makers need to understand unique characteristics of Hispanic communities to design strategies and interventions for equitable access to online health information among underserved populations. This understanding may lead governments to develop policies to allocate and disseminate infrastructures and resources [74,76]. Large-scale improvement in the Internet technology infrastructure will reduce costs and barriers to accessing health information [77]. To provide timely and accurate health information across populations, policy makers need to consider a broad spectrum of health literacy levels and cultural issues of the designated population [78].

### Conclusions

This is the first large-scale study of online HISBs in the Hispanic population. This study not only confirmed factors associated with online HISBs identified in the literature but also revealed findings that were not previously described. Also, this study showed that respondents’ demographic, situational, and literacy factors were significantly associated with their household members’ online HISBs. Studies of online HISBs need to consider various associated factors to explain behavior [79]. To increase the number of online health information seekers among Hispanics, cultural values such as familism need to be considered. Results of this study can provide a foundation for the development of informatics-based interventions to improve the health of Hispanics in the United States.

## Abbreviations

CCPH: Columbia-Community Partnership for Health
HINTS: Health Information National Trends Survey
HISB: health information seeking behaviors
NVS: Newest Vital Sign
SF-8: Short Form-8 survey
WICER: Washington Heights Inwood Informatics Infrastructure for Comparative Effectiveness Research
